# Gender Minority Stress and Health Perceptions Among Transgender Individuals in a Small Metropolitan Southeastern Region of the United States

**DOI:** 10.1089/trgh.2019.0028

**Published:** 2019-10-21

**Authors:** James A. Griffin, Tracy N. Casanova, Elizabeth D. Eldridge-Smith, Lara M. Stepleman

**Affiliations:** ^1^ LGBT Health Resource Center, Chase Brexton Health Care, Baltimore, Maryland.; ^2^ Department of Psychiatry and Health Behavior, Medical College of Georgia, Augusta University, Augusta, Georgia.

**Keywords:** barriers to care, gender minority stress, health disparities, transgender health

## Abstract

**Purpose:** Transgender individuals continue to face wide-ranging health disparities, which may be due in part to unique and chronic gender identity-related stressors. The present study assessed the relationships between barriers to health care, proximal minority stress related to perceived community safety, and overall health perceptions of transgender individuals living in a small metropolitan region of the Southern United States.

**Methods:** Participants included 66 transgender individuals who took part in a larger lesbian, gay, bisexual, transgender, and queer (LGBTQ) community needs assessment study. Participants completed measures of barriers to health care, inclusive of medical access barriers, psychosocial needs barriers, and personal resource barriers, perceptions of LGBTQ safety within the region, and overall perceptions of health.

**Results:** Results revealed that psychosocial needs barriers, personal needs barriers, and perceived lack of community safety were correlated with poorer self-perceptions of overall health, with psychosocial needs barriers and perceived lack of community safety independently predictive of poor health perceptions.

**Conclusions:** The study demonstrates the need for greater health resources and access to care, as well as improved community conditions for transgender individuals, particularly those in less populated, Southern regions of the United States, to improve health quality and ultimately reduce community health disparities.

## Introduction

Transgender individuals continue to experience an array of chronic health disparities as compared with cisgender individuals.^1^ While there is burgeoning literature on transgender specific health needs (e.g., gender transitioning) and specific critical medical conditions (e.g., HIV), less is known about general health concerns among transgender individuals compared with other groups. A large-scale review of 116 articles related to transgender health published between 2008 and 2014 identified general health (i.e., diabetes, cancer) as the least researched health-related category.^2^ Emerging research suggests that transgender individuals may experience worse overall health as compared with cisgender counterparts. For instance, a national survey of over 2000 transgender individuals found a higher burden of disability, mental and chronic health conditions among transgender respondents compared with cisgender respondents.^1^ Furthermore, transgender individuals were more likely to perceive their overall health as “poor/fair” compared with cisgender individuals. Such self-perceptions of health are often good proxies for various biological health markers (e.g., blood pressure, body mass index, metabolic and immune markers) in underserved populations,^3^ and are predictive of future disability and morbidity.^4^ The present study aims to fill a gap in the existing literature by examining factors related to health perceptions among transgender individuals in a Southern metropolitan region.

### Barriers to health care

Transgender health care is often substandard. The unique health needs of transgender individuals are often ignored by the medical community and prejudice remains commonplace, preventing transgender individuals from receiving competent care.^5^ Although transgender health care research has increased in recent years, the focus on barriers to health care for gender minorities has lagged. The National Transgender Discrimination Survey found a number of critical barriers to health care for transgender people, including denial of care by a provider, low rates of insurance coverage, verbal harassment from medical providers, and delay of seeking medical care due to fear of discrimination.^6^ Additionally, the survey results highlighted higher rates of HIV, smoking, drug and alcohol use, and suicide attempts among transgender individuals, compared with the general population.^6^ Discriminatory provider behaviors may stem from a lack of education among medical providers, as the curriculum offered at many medical schools in the United States typically includes limited information regarding transgender health.^7^ In addition to limited training, providers may act on implicit biases based on inaccurate stereotypes.^8^ As compared with cisgender persons, transgender individuals are especially vulnerable to discrimination and prejudice when seeking health care as transgender identities often cannot be concealed from providers who have access to their medical records.

Experiences of transgender discrimination and stigma have been found to negatively affect both physical and mental health.^8–10^ Transgender individuals experience various forms of discrimination in many aspects of life, such as in their local communities, in the workplace, and within the health care system.^6,11^ Transgender individuals report high rates of verbal, physical, and sexual harassment.^11,12^ They are also often denied work opportunities due to their gender identity, likely contributing to the economic hardships that are often endorsed by transgender individuals.^12,13^ Additional stress and discrimination comes from lack of access to basic needs, such as bathrooms and appropriate housing.^14^

### Minority stress theory

Meyer's minority stress theory proposes that unique stressors specific to minorities, such as identity-based discrimination and negative internalized evaluations of these discriminatory experiences, converge to contribute to poor mental and physical health.^15^ Meyer distinguishes three types of stressors that minority individuals face: general stress, distal minority stress, and proximal minority stress. General stress may include barriers to health care, such as those that disproportionately burden transgender individuals.^6^ Distal minority stressors are adverse experiences or reactions from the environment, including health care setting, related to the individual's minority identity.^15^ Distal stressors may occur at different levels, such as structural, interpersonal, and individual levels.^16^ Distal stressors can be either chronic or acute and are objective experiences as opposed to the subjective experience of proximal stressors. Proximal stressors include fear of discrimination and negative internalized beliefs regarding one's own group.^15^ Although this model was originally applied to sexual minorities, the minority stress theory has been applied to transgender experiences.^17^ More research is needed to explore this model in relation to the physical health of gender minority individuals.

External stressors (i.e., general and distal minority stress) attributed to one's identity not only have direct negative health consequences, but also give rise to proximal stressors that may be chronic sources of distress, according to minority stress theory.^15,17^ For instance, research has demonstrated links between transgender discrimination and harmful health behaviors such as suicidality and substance abuse.^18,19^ While experiences of minority stress appear to negatively affect health outcomes, few studies have specifically examined the direct link between proximal gender minority stress and health outcomes.

The geographic context of health care intersects with the negative health care experiences related to the transgender identities. For instance, individuals in the southern United States are less likely to be insured, have access to health care, and experience poorer reported overall health compared with other regions.^20^ The location of the current study, the Central Savannah River Area (CSRA), includes the moderately sized city of Augusta, GA, surrounded by mostly rural counties. According to the Human Rights Campaign, Augusta received a score of 35/100 on Healthcare Equality Index, demonstrating that Augusta is providing subpar care for the lesbian, gay, bisexual, transgender, and queer (LGBTQ) community.^21^ For the transgender community, Augusta received scores of 0/6 for inclusive health care benefits and 0/2 for trans-specific services offered in the community.

### The present study

This study explores the associations between gender minority stress and self-perceptions of health. Specifically, the study will examine external stressors of barriers to health care, including personal resource barriers, medical access barriers, and psychosocial barriers, and proximal minority stress in terms of perceptions of safety for LGBTQ individuals within the region. It is hypothesized that distal and proximal stressors will be positively correlated with, and independently predictive of, overall poor perceptions of health.

## Methods

### Participants

Participants of this study were recruited as part of a larger community health needs assessment conducted for LGBTQ individuals living in the CSRA. Participants were recruited in-person through LGBTQ community venues (e.g., bars) and events (e.g., Augusta Pride) and online through link provided on distributed flyers and business cards, as well as on the website for the Equality Clinic of Augusta, an all-volunteer free LGBTQ primary care clinic. Recruitment lasted from January through June of 2016. The study was granted exempt status by the Augusta University Institutional Review Board due to anticipated minimal to no risk of harm to participants of the study. Participants were provided electronic informed consent before participation in the electronically administered survey, to which they needed to agree before study participation. The consent document described the study purpose, the voluntary nature of their participation, and the expected risks and benefits of participation consistent with accepted ethical research practices. Participants did not receive direct monetary compensation for their participation in the study; however, they were offered the opportunity to enter into a raffle drawing for the chance to win a gift card or VIP pass to the Augusta Pride Festival upon completion of the study. More detailed participant recruitment methods have been described elsewhere.^22^

For the parent study, a total of 602 individuals accessed the study, whereas 436 had adequate data and met the parent study's inclusion criteria of being an adult, sexual and/or gender minority identified, and living in the CSRA. A total of 88 transgender participants were included in the larger study sample. The present study includes 66 transgender individuals from the parent study who completed all items for variables of focus in the present study. See Table 1 for a full breakdown of sample demographics. Of these 66 individuals, 22 (33.3%) identified as transgender men, 20 (30.3%) identified as transgender women, and 24 (36.4%) identified with a nonbinary transgender identity (e.g., genderfluid, genderqueer, agender). Participants were predominantly White (71.2%) with a mean age of 30.44 years (standard deviation [SD]=11.68). A large majority of participants (78.1%) had at least some college education. Just over half (50.9%) of participants reported an annual household income below $20,000.

**Table 1.** Sample Demographics (*N*=66)

Variables	Range	*M* (SD)	*n* (%)
Age (years)	18–75	30.33 (11.68)	
Race			
White			47 (71.2)
Black			5 (7.6)
Hispanic			5 (7.6)
Other			9 (13.6)
Gender identity			
Transgender men			22 (33.3)
Transgender women			20 (30.3)
Gender nonbinary			24 (36.4)
Education level			
<High school			1 (1.6)
Diploma/GED			13 (20.3)
Some college			30 (46.9)
Associate's degree			7 (10.9)
Bachelor's degree			7 (10.9)
Graduate degree			5 (7.8)
Doctoral/professional degree			1 (1.6)
Household income (USD)			
<10,000			12 (23.5)
10,000–14,999			10 (19.6)
15,000–19,999			4 (7.8)
20,000–29,999			2 (3.9)
30,000–30,999			3 (5.9)
40,000–49,999			6 (11.8)
50,000–74,999			7 (13.7)
75,000–99,999			4 (7.8)
100,000+			3 (5.9)

Valid percentages reported due to missing data for some variables.

GED, general education development; SD, standard deviation; USD, US dollars.

### Measures

#### Demographics

A series of demographic questions were asked to assess participant gender identity, age, race, household income, and highest level of education. Gender identity was assessed using a two-step process as recommended by Reisner et al.^23^ Participants were first asked “What biological sex is listed on your original birth certificate?” Options provided were “Female,” “Male,” “Intersex,” “Not sure,” and “Prefer not to answer.” This was followed by a question asking “Which best describes your current gender identity.” Options provided were “Female,” “Male,” “Intersex,” “Transgender-Female to Male,” “Transgender-Male to Female,” “Genderqueer,” or “Other, please specify,” the latter of which had a free response field. Participants whose gender identity was incongruent with sex assigned at birth were included in the present study. Participants whose transgender identity could not be established (e.g., selected “Prefer not to answer” to the sex at birth item and a current gender identity that did not indicate a transgender identity, such as “Female”) were not included in the sample.

#### Barriers to care

Distal minority stress was assessed by a measure of barriers to obtaining appropriate health care services. Barriers to health care for LGBTQ individuals was measured using an adapted version of a Barriers to Care Scale (BACS), a 12-item scale assessing health care barriers for people living with HIV.^24^ The adapted version of the original BACS that is used in the present study was created for the 2015 New York State LGBT Needs Assessment.^25^ This version modified items of the original scale to reflect LGBTQ-specific barriers to health care (e.g., “The lack of health care professionals who are adequately trained and competent in LGBT health care”). Items maintained the same four-point Likert scale, ranging from “Major problem” to “Not a problem at all” of the original BACS measure. Scale items were reverse coded so that participant scores reflect greater barriers to care.

The original BACS consisted of a four-factor structure of barriers to care: geography/distance, medical and psychological, community stigma, and personal resources. A principal component analysis with varimax rotation conducted on the adapted items used in the present study supported a three-factor structure. The first factor, medical access barriers, consists of four items inclusive of general access barriers (e.g., “Long distances to medical facilities and personnel”) and discriminatory practices (e.g., “Medical personnel [e.g., physicians, nurses], who discriminate against LGBT people when providing direct care”). The second factor, psychosocial needs barriers, consists of six items inclusive of lack of specific LGBTQ psychosocial resources (e.g., “The lack of psychological support groups for LGBT people”) and community stigma (e.g., “Community residents' stigma against LGBT people”). The third factor, personal resource barriers, consists of two items reflecting other general barriers to care (e.g., “My personal financial resources”). Scores for items within each subscale were averaged to derive a final subscale score, with higher scores reflecting greater barriers to health care. Internal reliability estimates for each subscale were adequate in the present study, with subscale Cronbach's alpha scores ranging from 0.67 to 0.88 (full scale *α*=0.86). For comparison, subscales demonstrated acceptable internal reliability (*α* range=0.72–0.78) with a total scale Cronbach's alpha of 0.86 in the initial validation study.

#### CSRA safety

Proximal gender minority stress was assessed using an item of perceived safety within the CSRA region. Perceived safety of the CSRA region for LGBTQ individuals was assessed with a single item, asking “How safe as an LGBT individual do you feel in the CSRA, Augusta, or surrounding areas?” Participants responded along a five-point Likert-type scale ranging from “Very safe” to “Very unsafe.” Higher scores reflected a greater lack of perceived safety for LGBTQ individuals in the CSRA region.

#### General health

The primary outcome of the study is general health self-perceptions. Participants responded to a single item of overall health taken from the Centers for Disease Control and Prevention's Health-Related Quality of Life self-report four-item measure (HRQOL-4).^26^ The item assessing general health perceptions asks, “Which best describes your general health?” Participants responded along a five-point Likert-type scale ranging from “Excellent” to “Poor,” with higher scores indicating poorer overall health perception.

### Data analyses

The present study employs a baseline, correlational design. Surveys were completed electronically, with an average completion time of 30 min. Responses were collected and stored electronically through Qualtrics until the study ended. Data were then cleaned and analyzed using SPSS software (version 25). In the present study, only barriers to care, CSRA safety, and general health perception items among transgender respondents were analyzed. Descriptive statistics of this sample were derived. Additionally, a series of Pearson correlational analyses were conducted to assess zero-order associations between predictor and criterion variables. A one-way analysis of variance (ANOVA) was conducted to test differences in general health perceptions across genders. A final hierarchical linear regression model was conducted predicting general health perceptions. Significant barriers to care were entered into Step 1 and proximal minority stress (i.e., CSRA safety) was entered into Step 2.

## Results

Descriptive analyses were conducted on the primary variables of focus in this study. The mean score for the study's primary outcome variable, health perception, was 2.67 (SD=0.97). The medical access barriers to health care scale has a mean score of 2.11 (SD=0.82), whereas the psychosocial needs barriers score had a mean of 1.02 (SD=0.89), and the personal resource barrier scale had a mean of 2.52 (SD=1.06). The mean CSRA safety score was 2.96 (SD=0.84). All five scales approximated normal distributions, based on indices of skewness and kurtosis, as well as examination of normality plots.

A series of bivariate correlational analyses were conducted to assess the associations between demographics, gender minority stress, and health perception variables. Table 2 displays the full results of these analyses. Barriers to care subscales were all significantly intercorrelated. Perceived lack of CSRA safety was moderately correlated with medical access and psychosocial needs barriers to care. No demographic variables were significantly associated with the primary outcome variable, health perception. Two barriers to care variables, psychosocial needs barriers and personal resource barriers, and proximal minority stress variable perceived lack of CSRA safety were significantly, moderately associated with health perceptions, such that as perceived lack of CSRA safety or barriers to health care increases, health perception was poorer.

**Table 2.** Correlations Between Health Perceptions, Demographics, and Gender Minority Stressors (*N*=66)

	1	2	3	4	5	6	7	8
1. Health perception	—							
2. Age	−0.062	—						
3. White	0.172	0.014	—					
4. Education level	−0.125	0.282^**^	0.225	—				
5. Medical access barriers	0.243	0.102	0.096	0.066	—			
6. Psychosocial needs barriers	0.405^**^	0.029	0.173	−0.062	0.504^**^	—		
7. Personal resource barriers	0.432^**^	−0.087	0.015	−0.301^*^	0.443^**^	0.267^*^	—	
8. Perceived lack of safety	0.342^**^	−0.112	0.368^**^	0.083	0.393^**^	0.355^*^	0.128	—

^*^ *p*<0.05; ^**^p<0.01.

Point biserial correlations reported where one variable is dichotomous. Pearson correlations reported where both variables are continuous.

A one-way ANOVA was conducted to test differences in general health perceptions across three gender groups: transgender men, transgender women, and gender nonbinary. Results revealed no statistically significant differences in health perceptions across gender groups (*F*_(2,63)_=0.06, *p*=0.99). Given no effect of gender on health perception at the bivariate level, gender was not included in the final regression model predicting health perception.

A multiple linear regression model was conducted to assess the independent contributions of barriers to care and proximal stress variables significant at the bivariate level to the variance in health perception scores. Table 3 displays the full results of this analysis. Since no demographic variables were significantly associated with the main outcome variable, health perception, they were omitted from the model. Barriers to care variables (psychosocial needs barriers and personal resource barriers) were entered together in Step 1 of the regression model. At this step, psychosocial needs barriers emerged as a significant, independent predictor of health perception when accounting for the effects of personal resource barriers. Proximal minority stress variable perceived lack of CSRA safety was entered into Step 2 of the model. At this step, psychosocial needs barriers remained independently predictive of health perceptions, and lack of CSRA safety emerged as an independent predictor of health perceptions when accounting for the effects of distal stress variables. The final model accounted for 27% of the variance in health perceptions.

**Table 3.** Multiple Linear Regression Model of Gender Minority Stress Variables Predicting Health Perceptions (*N*=62)

Variables	Step 1		Step 2	
	*β*	95% CI	*β*	95% CI
1. Psychosocial needs barriers	0.339^**^	0.208 to 1.907	0.249^*^	0.004 to 0.534
2. Personal resource barriers	0.233	−0.004 to 0.427	0.224	−0.007 to 0.412
3. Perceived lack of safety			0.259^*^	0.024 to 0.575

^*^ *p*<0.05, ^**^p<0.01.

Model Summary: Step 1 *R*^2^=0.212, *F*_(2,60)_=8.050, *p*<0.05; Step 2 *R*^2^=0.270, *F*_(3,59)_=7.283, *p*<0.05.

CI, confidence interval.

## Discussion

The present study aimed to assess external gender minority stressors as related to health care experiences and proximal minority stress as related to perceptions of community safety associated with health perceptions among transgender individuals living in the CSRA of Georgia and South Carolina. It was hypothesized that health care-related stressors, or barriers to care, and proximal minority stress of perceived community safety would predict poor health perceptions. Results revealed that psychosocial needs barriers to care, which include lack of mental health support and community stigma, and personal resource (e.g., financial) barriers to care were associated with poorer health perceptions, with psychosocial needs barriers being independently predictive when accounting for other minority stress variables. Perceptions of lack of safety for LGBTQ individuals was also independently associated with poorer health perceptions. These findings are consistent with Meyer's original minority stress theory,^15^ as well as subsequent theoretical models extending Meyer's work to gender minority individuals.^17^

### Study implications

This study adds to a growing body of literature demonstrating the impact of gender minority stress experiences on the health of transgender individuals. With poorer overall health for transgender individuals in the South as compared with other regions,^20^ general health perceptions served as the target outcome variable in this study. Health perceptions, while a subjective assessment of health, have been shown to be a good indicator of actual physical health.^3^ For transgender individuals in smaller metropolitan areas of the South, experiences of psychosocial barriers, such as lack of appropriate psychological support and community stigma, and internalized beliefs regarding community safety are related to poorer health perceptions, and thus are likely to contribute to health disparities experienced by this group. These findings are consistent with prior research demonstrating effects of transgender minority stress on health outcomes.^9,10^

Efforts to reduce health disparities for transgender individuals should be multifaceted and must include a focus on the reduction of minority stress experiences. This focus should happen at interpersonal, institutional, community, and broader societal levels. At the interpersonal level, access to sensitive and competent health care, including appropriate mental health services, is critical. Models of appropriate medical care and mental health care for transgender persons exist^5,27^; however, transgender individuals often lack access to transgender-sensitive and competent care.^6^ It should be incumbent upon academic and medical training programs to adequately train providers in appropriate transgender health care delivery and to assist providers in examining their inherent biases as they relate to transgender individuals.^8^

At institutional and community levels, formal policies are needed to provide for the protection of transgender individuals' rights and dignity. While this is critical for health care agencies and systems, with current accepted standards of practice in existence,^28^ this should extend to other nonhealth care-related community institutions, as well. At the community level, greater transgender visibility, such as gender minority inclusive marketing, community issue awareness, and community support, is needed to combat stereotypes and stigma to reduce the occurrence of enacted stigma against transgender individuals. Additionally, greater community resources should be directed toward transgender health and emotional support needs, particularly in smaller, under-resourced regions such as the CSRA.^20,21^ Finally, policy at the local, state, and federal levels are needed to protect transgender rights and direct resources to eliminate health disparities experienced by transgender individuals.

### Study limitations

Several limitations exist for the present study. Some of these limitations concern construct measurement in this study. Minority stress experiences were not exhaustive and limited to experiences of barriers to health care services and perceptions of LGBTQ community safety. The BACSs consisted of a mix of general barriers as well as distal minority stressors, such as discrimination and stigma that could not be parsed apart. Moreover, proximal minority stress and health perceptions were assessed by single item measures. While minority stress measures assess common experiences of transgender individuals, not all items specifically assess the extent to which the experience is based on their transgender identity. Items also do not assess transgender specific health care needs, such as access to gender-affirming medical care (e.g., hormone replacement therapy). Future studies should expand their scope to include other minority stress experiences, such as discrete experiences of discrimination and internalized transphobia. Additionally, a subjective indicator of health, perceptions of health, was assessed in the present study as opposed to an objective measure.

There are additional, broader limitations to consider when interpreting and applying the results of this study. Since the study was correlational in design, causality cannot be determined based on the present study's results. Longitudinal study designs are needed to assess the effects of minority stress experiences on health over time. Finally, the sample size was small and restricted to transgender individuals in the CSRA region of the Southeastern United States. The sample was of transgender individuals who are likely well connected to the community given sampling procedures. These results may not accurately reflect experiences of transgender individuals in different contexts. For instance, those who are not well connected to their local LGBTQ communities may have different stress and health-related experiences. Future research should expand on the findings of the current study while addressing the study's limitations.

## Conclusion

This study assessed gender minority stress experiences related to health perceptions among transgender individuals in a small metropolitan area of the South. Overall, psychosocial needs barriers, resource barriers, and perceptions of lack of safety for the LGBTQ community were indicative of poorer health self-perceptions, which may relate to known health disparities. To reduce health disparities for transgender individuals, efforts should focus on combating stigma from interpersonal to systemic levels, while also increasing access to resources and community visibility. Additional research is needed to determine causal mechanisms between minority stress experiences and health disparities affecting the transgender community.

## Abbreviations Used

ANOVA: analysis of variance
BACS: Barriers to Care Scale
CSRA: Central Savannah River Area
LGBTQ: lesbian, gay, bisexual, transgender, and queer
SD: standard deviation
